# *Trichospirura aethiopica* n. sp. (Nematoda: Rhabdochonidae) from *Malacomys longipes* (Rodentia: Muridae) in Gabon, first record of the genus in the Ethiopian Realm

**DOI:** 10.1051/parasite/2012004

**Published:** 2013-01-31

**Authors:** Odile Bain, Kerstin Junker

**Affiliations:** 1 Muséum National d’Histoire Naturelle, Parasitologie comparée, UMR 7205 CNRS CP 52 61 rue Buffon 75231 Paris Cedex 05 France; 2 ARC-Onderstepoort Veterinary Institute, Parasites, Vectors and Vector-borne Diseases Programme, Private Bag X05 Onderstepoort 0110 South Africa

**Keywords:** *Trichospirura aethiopica* n. sp., Rhabdochonidae, Nematoda; *Malacomys*, Rodent, Gabon

## Abstract

*Trichospirura aethiopica* n. sp. is described from unidentified tubular structures (pancreatic ducts?) near the stomach of the murid *Malacomys longipes* Milne-Edwards, 1877 in Gabon. The extremely long and narrow buccal capsule, posterior position of the vulva, unequal spicules and absence of caudal alae readily identified the specimens as belonging to *Trichospirura* Smith & Chitwood, 1967, but a combination of several characters distinguished them from the described species in this genus. Males of the new species are characterized by the absence of precloacal papillae, the presence of four pairs of postcloacal papillae and a left spicule length of 165–200 μm. With only five nominal and one unnamed species, the host range of *Trichospirura* extends into the Neotropical, Indo-Malayan and Ethiopian Realms and comprises three classes of vertebrates, Amphibia, Reptilia and Mammalia, suggesting a larger species diversity than that currently recorded. Detection is difficult as predilection sites are often outside the gut lumen. It was noted that, irrespective of their geographic origin, species from mammals share certain characters (shorter left spicule and absence of precloacal papillae) that oppose them to those from amphibians and reptiles. A hypothesis for the origin of *Trichospirura* in mammals through a remote host-switching event in tupaiids in southern Asia, likely facilitated by the intermediate hosts, and for their subsequent migration to the Ethiopian and finally Neotropical Realm is proposed. Regarding the two species from anurans and saurians in the Antilles, one or two host-switching events are considered equally possible, based on morphological characters.

## Abstract (fr)

*Trichospirura aethiopica* n. sp. est décrit de spécimens infestant des structures tubulaires non identifiées (canaux pancréatiques ?) près de l’estomac du Muridae *Malacomys longipes* Milne-Edwards, 1877 provenant du Gabon. La capsule buccale étroite et extrêmement longue, la position postérieure de la vulve, les spicules inégaux et l’absence d’ailes caudales identifient clairement les spécimens comme appartenant à *Trichospirura* Smith & Chitwood, 1967, mais une combinaison de plusieurs caractères les distingue des espèces déjà décrites dans le genre. Les mâles de la nouvelle espèce sont caractérisés par l’absence de papilles précloacales, la présence de quatre paires de papilles postcloacales et un spicule gauche long de 165–200 μm. Avec seulement quatre espèces nominales et une espèce non nommée, les hôtes de *Trichospirura* vivent dans les domaines biogéographiques néotropical, indo-malais et éthiopien et appartiennent à trois classes de vertébrés, amphibiens, reptiles et mammifères, ce qui suggère une diversité d’espèces plus grande que ce qui est actuellement connu. La détection est difficile parce que les sites d’infestation sont souvent en dehors de la lumière digestive. Nous avons noté que les espèces parasites de mammifères, quelle que soit leur origine géographique, partagent certains caractères (spicule gauche plus court et absence de papilles précloacales) qui les opposent à celles des amphibiens et reptiles. Une hypothèse de l’origine de *Trichospirura* chez les mammifères est proposée, incluant un événement de changement d’hôte lointain chez les Tupaiidae en Asie du sud-est, probablement facilité par les hôtes intermédiaires, et leur migration ultérieure vers le domaine éthiopien et finalement néotropical. En ce qui concerne les deux espèces d’anoures et de sauriens des Antilles, une ou deux événement de changements d’hôtes sont considérés comme également possibles sur la base des caractères morphologiques.

## Introduction

Representatives of the large family Rhabdochonidae Travassos, Artigas & Pereira, 1928 generally parasitize fishes, except for those of one genus, *Trichospirura* Smith & Chitwood, 1967 (= *Freitasia* Baruš & Coy Otero, 1968; [12]), which are found in tetrapod vertebrates. Reports of *Trichospirura* species are rare. Their infection sites are the small intestine, as in most of the rhabdochonids, or are unusual, such as the pancreatic or salivary ducts, or the abdominal cavity, in which the worms are encapsulated. Only four nominal and one unnamed species are recognized in the genus (Tables 1 and 2). This contrasts with the wide host range and geographic distribution of *Trichospirura*. Three species occur in South and Central America and are parasites of platyrrhinian monkeys [16], of saurians [2] and of anurans [13]; the others are from Malaysia, where they parasitize tupaiid insectivores and chiropterans [5]. The remarkable host range and geographic distribution of *Trichospirura* are further extended by the discovery of a new species from an African murid. This poses the question of its origin.

**Table 1.** Morphological characteristics of the males of *Trichospirura aethiopica* n. sp. from *Malacomys longipes* in Gabon and *Trichospirura* spp.

Species	*aethiopica* n. sp.			*leptostoma*	*willmottae*	*teixeirai*				*amphibiophila*
Authority and date	–			Smith & Chitwood, 1967	Chabaud & Krishnasamy, 1975	(Baruš & Coy Otero, 1968) Moravec, 1975				Moravec & Kaiser, 1994
Reference, if different from authority and date	Present study			–	–	–	Coy Otero, 1970	Coy Otero & Baruš, 1979	Moravec & Puylaert, 1970	–
Specimen number	Holotype	Paratype 1	Paratype 2	–	Holotype	Holo- & Paratype	–	*n* = 10	*n* = 1^e^	Holotype
Body length (mm)	13.4	11.7	11.25	10.8–15	7	10.04 & 10.52	10.04–10.52	9.3–13.62	–	6.24
Body width at mid-body	205	190	–	180	170	230 & 270	230–270	200–260	–	122
Nerve ring to apex	375	350	330	–	195	680 & 780	680–780	770–820	950	366
Deirids to apex	325	390	280	–	225	–	–	Absent	ND	222
Excretory pore to apex	–	–	–	–	300	860^c^	–	350–390	500	456
Buccal capsule length	500	425	490	–	380	620 & 780	620–780		850	480
Oesophagus total length	1475	1790	1700	–	1220	1260 & 1280	1050	880–1100	–	1161
Muscular oesophagus length	460	450	420	–	250	210 & 230	210–230	200–270	–	225
Tail length	250	270	270	350^a^	160	220 & 230	220–230	140–190	–	123
Tail width at anus	90	90	80	105^a^	80^a^	110 & 120	–		–	65
Left spicule length	175	165	200	190	150	470 & 510	470–510	730–800	–	459
Handle of left spicule	60	32	–	50^a^	–	75^a^	–	–	–	92
Right spicule length	96	82	72	90	80	97	97	88–120	–	90
Pairs of precloacal papillae	0	0	0	0	0	1	1	1^d^	–	2
Pairs of postcloacal papillae	4	4	4	5 to 6	5 (4 + 1)^b^	3	3	4	–	5
Type host	*Malacomys longipes*			*Callithrix* (*Callithrix*) *jacchus*	*Tupaia glis*	*Anolis equestris*				*Eleutherodactylus martinicensis*
Authority and date	Milne-Edwards, 1877			Linnaeus, 1758	Diard, 1820	Merrem, 1820				Tschudi, 1838
Host family	Muridae			Cebidae	Tupaiidae	Polychrotidae				Eleutherodactylidae
Site of infection	Tubes near stomach			Pancreatic ducts	Salivary duct	Intestine				Abdominal cavity^f^
Geographic origin	Gabon			Brazil	Malaysia	Cuba				French Antilles

a measured on figures;

b four pairs in anterior mid-part of tail and a subterminal pair;

c According to Moravec & Puylaert (1970) this figure is incorrect and the excretory pore is situated more anteriorly;

d sometimes absent;

e measured on Figure 2G;

f encapsulated near liver.

Measurements in micrometres unless otherwise specified.

**Table 2.** Morphological characteristics of the females of *Trichospirura aethiopica* n. sp. from *Malacomys longipes* in Gabon and *Trichospirura* spp.

Species	*aethiopica* n. sp.		*leptostoma*	*willmottae*	sp.^d^	*teixeirai*^e^	*amphibiophila*
Specimen status or reference	Allotype	Paratype	Smith & Chitwood, 1967	Chabaud & Krishnasamy, 1975^c^	Chabaud & Krishnasamy, 1975^c^	Coy Otero, 1970	Moravec & Kaiser, 1994
Body length (mm)	13.4	15.6	12–20	7.5	5.6	9.88–19	10–11.18
Body width at mid-body	225	255	350	230	70	240–440	340
Nerve ring to apex	320	330	330	225	230	750–990	411
Deirids to apex	270	285	240	190	180	–	216–225
Excretory pore to apex	430	370	650	290	290	825^a^	495
Buccal capsule length	460	420	470–500	340	340	660–880	765
Buccal capsule width	9	8	20^a^	8^a^	–	10–16	9
Oesophagus total length	1360	1600	1730	1380	795	1180–1390	1358
Muscular oesophagus length	330	400	380	230	155	230–260	270
Distance vulva to posterior extremity	450	460	620	240	280	450–610	360–435
Vagina length	100	–	–	–	–	–	–
Ovijector length (including vagina)	300	350	1650 & 1350^b^	300^a^	130	360–450	–
Tail length	250	280	330	140	170	180–290	233–261
Tail width at anus	60	70	100^a^ & 70^b^	75^a^	35^a^	–	80^a^
Egg size	50–55 × 28	48 × 22	50 × 25	50 × 32	–	47–49 × 20–24	51–54 × 24–27

a measured on drawings;

b in the present study;

c Chabaud & Krishnasamy (1975);

d immature specimen;

e from four host species, including *Anolis equestris*, the type host species originally infected with males only.

Measurements in micrometres unless otherwise specified.

## Materials and methods

**Figure 1.**
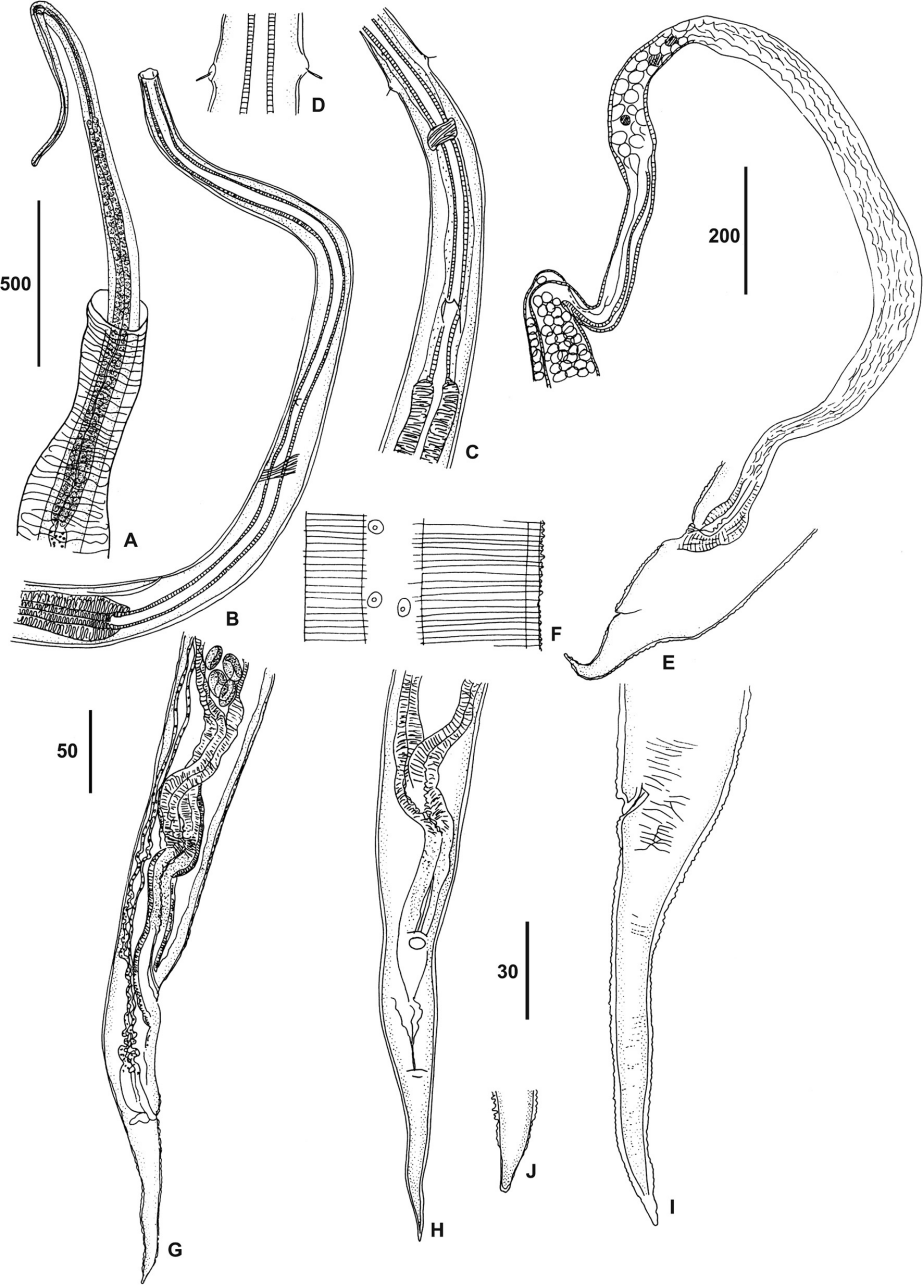
*Trichospirura* spp. females. **A**, *T. aethiopica* n. sp. Anterior region of a worm half dissected from a tube with an external muscular layer, note the anterior bend. **B**, part of buccal capsule and beginning of muscular oesophagus. **C**, detail of nerve ring, excretory pore and deirids, ventral view. **D**, at level of deirids, ventral view. **E**, *T. leptostoma*, posterior extremity, ovijector with a dilated chamber and uteri. **F–I**, *T. aethiopica* n. sp. **F**, detail of the cuticular sheath and lateral chord. **G, H**, posterior part, ovijector and uteri, right lateral and ventral view, respectively. **I**, tail, left lateral view (cuticular sheath and striae drawn at level of anus). **J**, caudal extremity, ventral view. Scales in μm: A, E, 500; B, C, F, I, J, 50; D, 30; G, H, 200.

In 1996 Dr. G. Dubreuil, Centre International de Recherches Médicales de Franceville, captured rodents in Gabon for virology research. Several animals were fixed in formalin and sent to the Muséum National d’Histoire Naturelle (MNHN), Paris, France, to augment both the collection of mammals and that of zooparasitic nematodes. In a Big-eared swamp rat, *Malacomys longipes* Milne-Edwards, 1877, rhabdochonid nematodes were recovered during the dissection of tissues near the stomach; several worms were found in the lumen of unidentified tubular structures; these did not seem to be granulomatous reactions of the host, because they possessed a regular wall with an external muscular layer (Figure 1A); they might have been pancreatic ducts.

For comparison with the new material, a female specimen of *T. leptostoma*, USNPC 61802, from *Callithrix* (*Callithrix*) *jacchus* (Linnaeus, 1758) and one male specimen of *T. amphibiophila* from *Eleutherodactylus martinicensis* (Tschudi, 1838), Institute of Parasitology, Academy of Sciences of the Czech Republic, České Budějovice, Helminthological Collection, No. N-602, were studied.

All specimens were cleared in lactophenol and examined under a Wild compound light microscope equipped with a drawing tube. Measurements were taken from drawings and are given in micrometres unless otherwise specified. The ovijector length was measured from the vulva to the division of the uteri and includes the vagina, as this structure was not identified in previously described species and there is no clear distinction between vagina and ovijector. In the description, the term buccal capsule, instead of pharynx or vestibule, is used for the tube between the mouth and the oesophagus in order to be consistent with other groups of nematodes.

The nomenclature and classification of small mammals follows Wilson & Reeder [20], that of anurans Frost [8] and that of reptiles Uetz [19]. The classification of biogeographic Realms follows Udvardy [18].

## *Trichospirura aethiopica* n. sp.

urn:lsid:zoobank.org:act:51435EEF-0EF7-49FE-8158-8B71D9EC43E2

  Type-host: *Malacomys longipes* Milne-Edwards, 1877.
  Type-locality: Makokou, 0° 34′ 00″ N, 12° 52′ 00″ E, Gabon.
  Collection date: 1996.
  Site of infection: tissular tubes near the stomach (pancreatic ducts?).
  Type-material: male holotype and two male paratypes, female allotype and a female paratype, Muséum National d’Histoire naturelle, Paris, MNHN 184SE.
  Prevalence and intensity: five worms in a single host.
  Etymology: the new species is named after its geographic origin, the Ethiopian Realm.

### Description (Figures 1–3; Tables 1 and 2)

**Figure 2.**
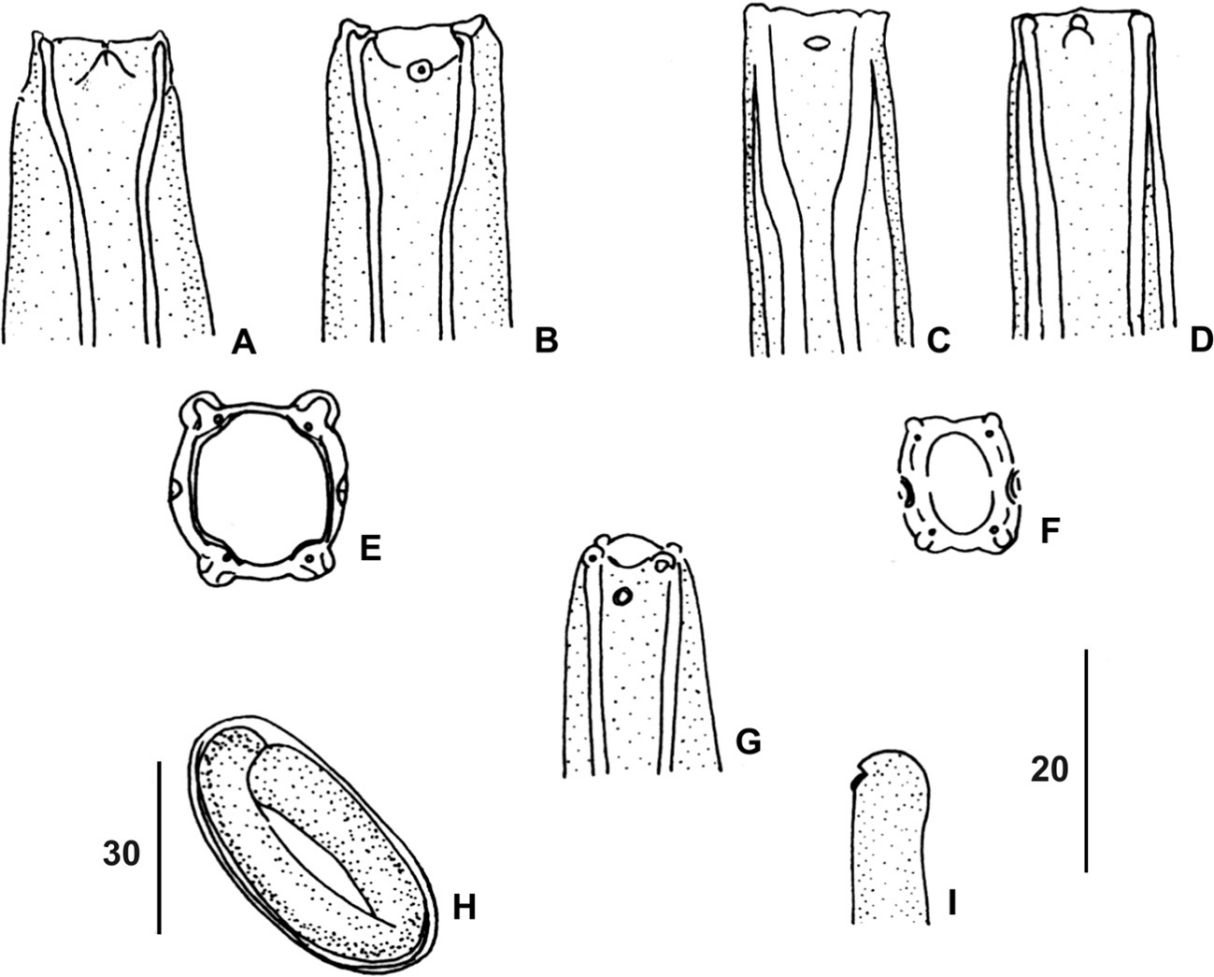
Anterior extremities of *Trichospirura* spp. **A, B**, *T. aethiopica* n. sp., female, submedian and lateral view, respectively. **C, D**, *T. leptostoma*, lateral and submedian view, respectively. **E, F**, en face view of *T. aethiopica* n. sp. **(E)** and *T. leptostoma* **(F). G**, *T. amphibiophila*, lateral view. **H, I**, *T. aethiopica* n. sp. **H**, larvated egg. **I**, cephalic extremity of first stage larva, dorsal view. Scales in μm: A, B, C, D, F, G, I, 20; H, 30. E, free hand sketch.

**Figure 3.**
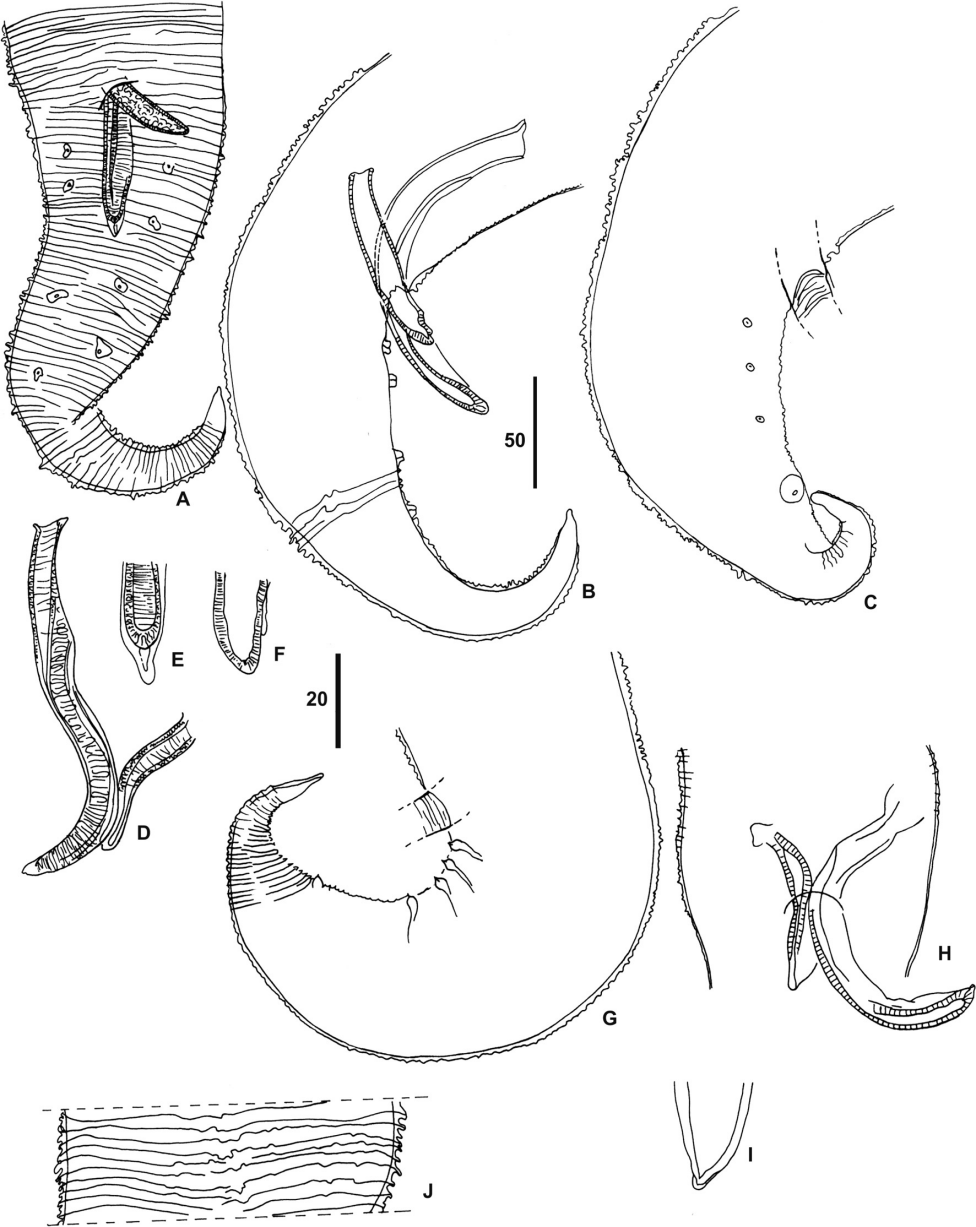
*Trichospirura* spp. males. **A–F**, *T. aethiopica* n. sp. **A, B**, posterior part, ventral and right lateral view, respectively. **C**, posterior part of another male. **D**, left spicule and distal part of right spicule, ventral view. **E, F**, distal extremity of the left and the right spicule, respectively. **G–J**, *T. leptostoma*; **G**, posterior part, left lateral view. **H**, spicules, ventral view. **I**, distal extremity of the right spicule. **J**, cuticular sheath at mid-body, ventral view. Scales in μm: A–D, G, H, 50; E, F, I, 20.

External layer of body cuticle forming a thin sheath with regular transverse salient crests (Figures 1F, I and 3A). Very thin anterior part, with acute bend at level of distal part of buccal capsule. Body attenuated posteriorly from vulva to tip of tail. Lateral chords narrow; excretory canals not noticed. Excretory pore slightly anterior to proximal end of muscular oesophagus. Nerve ring surrounding buccal capsule in posterior quarter and anterior to excretory pore. Deirids spindle-shaped, generally anterior to nerve ring. Head (Figure 2A, B, E): four groups of latero-median papillae; each group composed of a small but obvious external labial papilla, situated on the internal aspect of the mouth and an external salient cephalic papilla. Depressed amphidial aperture posterior to head papillae (Figure 2A, B, E). Mouth as wide as apex of body, almost square, with rounded angles (Figure 2E). Extremely long and thin buccal capsule, well-sclerotized, hardly dilated at anterior end or not at all, depending on orientation (Figure 2A, B); no «muscular ring» (see Moravec & Puylaert [14]) identified in posterior part of buccal capsule.

Female (Figure 1): didelphic, prodelphic. Vulva preanal and depressed. Ovijector: straight vagina with short vagina vera, followed by a part with thick granulous epithelium and thin external layer of muscles. Two uteri, each beginning with a short narrow part with thick muscular walls, subsequently widening into thin-walled tubes containing embryonated eggs (Figure 1G, H). Tail long, thin, with conical tip, blunt in ventral view; anus slightly depressed. Eggs thick-shelled, containing larva with left, subterminal, well-sclerotized hook (Figure 2H, I).

Male (Figure 3): tail attenuated in distal part, extremity pointed or blunt. Four ventro-lateral pairs of caudal papillae, all postcloacal, rather regularly distributed, some occasionally larger (Figure 3A–C). Spicules thick. Left spicule with short handle and three-times longer lamina with narrow latero-ventral membrane; distal end lined with narrow membrane. Right spicule shorter, with blunt tip (Figure 3B, D–F). No gubernaculum.

### Taxonomic discussion

The specimens recovered from *M. longipes* in Gabon display the typical characters of the rhabdochonid *Trichospirura*, namely the extremely long and narrow buccal capsule, posterior vulva, unequal spicules and absence of caudal alae [16]. In the two known species examined, three morphological features that went unnoticed in the original descriptions are similar to the material studied herein: the anterior body is often more or less abruptly bent and the vulva is depressed; in addition, a cuticular sheath forming transverse crests was observed in *T. leptostoma* (Figure 3J) and *T. amphibiophila*; it can also be identified on figures of *T. willmottae*, but was described as “annelures transversales” [5]; it might be a shared character, although it has not been reported in *T. teixeirai*. Despite these similarities, the material described herein can be distinguished from the currently known species of *Trichospirura* by the following characters (Tables 1 and 2).

*Trichospirura teixeirai*: parasitizes Polychrotidae (Sauria) in Central America; infection site intestine; longer buccal capsule, deirids absent [7], nerve ring at level of muscular oesophagus [2], excretory pore well anterior to nerve ring [7, 14], muscular ring present in posterior region of buccal capsule [14]; male tail with ventral longitudinal crests and strongly attenuated extremity, one pair of precloacal papillae and only three pairs of postcloacal papillae [2], instead of four in the present material (Coy Otero & Baruš [7] report four pairs of postcloacal papillae); two to three times longer left spicule, alae of lamina of left spicule wider [2, 6], a gubernaculum-like formation is present [7, 14].

*Trichospirura amphibiophila*: parasitizes Eleutherodactylidae (Anura) in Central America; infection site an abdominal cyst near the liver; excretory pore situated farther anteriorly; buccal capsule longer; two to three times longer left spicule; two pairs of precloacal and five pairs of postcloacal papillae [13].

The following two species from mammals have spicules similar in length and shape to the specimens studied herein and their caudal papillae are all postcloacal as well, but they are distinct in the following characters:

*Trichospirura leptostoma*: parasitizes Cebidae (Primates) in Brazil; infection site pancreatic ducts; vulva less posterior, ovijector (described herein) three times longer, thin-walled and dilated to form a pouch (Figure 1E); five or six pairs of postcloacal papillae in the male [16].

*Trichospirura willmottae*: parasitizes Tupaiidae (Scandentia) in Malaysia; infection site salivary ducts; body length of both sexes twice shorter; males with five pairs of postcloacal papillae, one being near the tail tip. Chabaud & Krishnasamy [5] described large transparent cells in the wall of the distal part of the ovijector.

*Trichospirura* sp. of Chabaud & Krishnasamy, 1975: parasitizes Vespertilionidae (Chiroptera) in Malaysia; infection site intestine; immature females, which are the only sex known, are much smaller with regard to all measurements presented in Table 2.

The material described from *M. longipes* represents a new species, for which the name *Trichospirura aethiopica* is suggested.

## Discussion

With only five nominal and one unnamed species, the rhabdochonid genus *Trichospirura* extends into three Realms, Neotropical, Indo-Malayan and Ethiopian, and its members parasitize three classes of vertebrates, Amphibia, Reptilia and Mammalia. The intestinal *T. teixeirai* was reported from several lizards, polychrotids, tropidurids and a gekkonid [6, 13]. *Trichospirura amphibiophila* was found in a single species of *Eleutherodactylus* only, although this eleutherodactylid host genus is highly diversified in Central America and the Antilles [13]; it might be an accidental infection from an unknown host, as the worms, one male and two females, were encapsulated in the abdominal cavity. *Trichospirura leptostoma* from the pancreatic ducts of the cebid *C.* (*C*.) *jacchus*, was discovered in the Texan laboratory to which these animals had been transported after they had been captured in two widely separated areas in Brazil, the southeastern Tupi Forest area and a vast area north of the Amazon; several animals were examined after approximately one to 16 months in captivity, likely hyperinfected through cockroaches, which were later shown to be the intermediate hosts in animal houses [1, 10]; a single specimen was also found in another cebid, *Saguinus oedipus* (Linnaeus, 1758), in Colombia [16]. The remaining three species were reported only once. *Trichospirura willmottae* was found in the salivary duct of a single *Tupaia glis* (Diard, 1820), *Trichospirura* sp. in the intestine of a single *Myotis mystacinus* (Kuhl, 1817) and *T. aethiopica* n. sp. in tubes near the stomach (pancreatic ducts?) of a single *M. longipes.* The probability of finding these worms is lowered by the fact that their infection sites are often outside the gut lumen.

When Baruš & Coy Otero [2] created *Freitasia*, they were unaware of the work of Smith & Chitwood [16]. Moravec [12] considered *Freitasia* a junior synonym of *Trichospirura*, while at the same time Chabaud [3] differentiated *Freitasia* from *Trichospirura* based on the shape of the buccal capsule, the former having a «pharynx dilated anteriorly to form a well-defined buccal capsule», the latter having a «pharynx not or only slightly dilated anteriorly». However, this character does not differ clearly between the two genera [2, 16]. It was nevertheless noted during this study that the parasites of mammals can be opposed to the two species from saurians and anurans from Central America (Antilles). Compared to *T. teixeirai* and *T. amphibiophila*, the species from mammals have a two to three times shorter left spicule, and precloacal papillae are absent whatever the Realm, Neotropical, Indo-Malayan or Ethiopian, and whatever the host order, Scandentia, Rodentia or Primates (Chiroptera are excluded because the male parasite is not known).

It is generally accepted that the genus *Trichospirura* was derived through host-switching from *Rhabdochona* Railliet, 1916, parasites of freshwater fishes [4, 5, 7, 13], and that the necessary adaptations were accompanied by an extraordinary lengthening of the buccal capsule. Excepting Australia, *Rhabdochona* has a worldwide distribution. Therefore host-switching might have occurred in each Realm. However, the characters highlighted in this study might suggest a lineage for the parasites of mammals from a remote host-switching event that occurred in Tupaiidae in southern Asia. Murids, which originated in this region, would have been infected before they reached Africa in successive waves during the Miocene, when the two continental masses were joined, approximately 15–11 Mya [9, 17, 21]. *Trichospirura leptostoma*, with a derived character (ovijector dilated into a chamber), might have originated from the same area, followed by a migration to Africa and finally to the Neotropical region, together with their primate hosts, the platyrrhine monkeys. Indeed, after decades of controversy, it is now well supported that the South American monkeys arrived from Africa by transoceanic migrations in a period (approximately 37–16.8 Mya; [15]) that overlaps with the Miocene era. In the Antilles, the two species from cold blooded vertebrates, a saurian and an anuran host, differ from each other in several important characters: nerve ring at the level of the muscular oesophagus or buccal capsule, excretory pore anterior [14] or posterior to nerve ring, deirids absent or present, gubernaculum present or absent, respectively. A single or two events of host-switching are equally possible.

Host-switching from fishes to other classes of vertebrates was likely facilitated by the intermediate hosts. They are aquatic arthropods, mainly mayflies, for the species of *Rhabdochona* (first life cycle elucidated by Moravec [11]) and cockroaches for *T. leptostoma* under experimental conditions [10]. No further life cycles have been studied to date [1]. Hosts that are parasitized by *Trichospirura* species are insectivorous. This also applies to *M. longipes*, the host of the new species, which, in addition, lives in an aquatic environment.
